# Assessing temporal processing of facial emotion perception with transcranial magnetic stimulation

**DOI:** 10.1002/brb3.136

**Published:** 2013-03-26

**Authors:** Yuri Rassovsky, Junghee Lee, Poorang Nori, Allan D Wu, Marco Iacoboni, Bruno G Breitmeyer, Gerhard Hellemann, Michael F Green

**Affiliations:** 1 Department of Psychology and Gonda Multidisciplinary Brain Research Center, Bar-Ilan UniversityRamat-Gan, Israel; 2 Department of Psychiatry and Biobehavioral Sciences, UCLA Semel Institute for Neuroscience and Human BehaviorLos Angeles, California; 3 Department of Neurology, University of CaliforniaLos Angeles, California; 4 Ahmanson-Lovelace Brain Mapping Center, University of CaliforniaLos Angeles, California; 5 Department of Psychology, University of HoustonHouston, Texas; 6 Department of Veteran Affairs, VISN-22, Mental Illness Research Education Clinical CenterLos Angeles, California

**Keywords:** Affect perception, facial emotion, transcranial magnetic stimulation, visual masking

## Abstract

The ability to process facial expressions can be modified by altering the spatial frequency of the stimuli, an effect that has been attributed to differential properties of visual pathways that convey different types of information to distinct brain regions at different speeds. While this effect suggests a potential influence of spatial frequency on the processing speed of facial emotion, this hypothesis has not been examined directly. We addressed this question using a facial emotion identification task with photographs containing either high spatial frequency (HSF), low spatial frequency (LSF), or broadband spatial frequency (BSF). Temporal processing of emotion perception was manipulated by suppressing visual perception with a single-pulse transcranial magnetic stimulation (TMS), delivered to the visual cortex at six intervals prior to (forward masking) or following (backward masking) stimulus presentation. Participants performed best in the BSF, followed by LSF, and finally HSF condition. A spatial frequency by forward/backward masking interaction effect demonstrated reduced performance in the forward masking component in the BSF condition and a reversed performance pattern in the HSF condition, with no significant differences between forward and backward masking in the LSF condition. Results indicate that LSF information may play a greater role than HSF information in emotional processing, but may not be sufficient for fast conscious perception of emotion. As both LSF and HSF filtering reduced the speed of extracting emotional information from faces, it is possible that intact BSF faces have an inherent perceptual advantage and hence benefit from faster temporal processing.

## Introduction

Extracting emotional information from faces is essential for adaptive functioning (Dolan 2002; Adolphs 2003; Erickson and Schulkin 2003). Given the importance of this ability for survival and normative functioning, emotional stimuli are thought to gain rapid and privileged access to specialized subcortical and cortical brain regions (Kanwisher et al. 1997; Ishai et al. 1999; LeDoux 2003, 2012; Rudrauf et al. 2008; Mitchell and Greening 2012). It is generally thought, for example, that basic facial expressions are automatically processed by the amygdala, with frontoparietal structures being involved in higher order processing, allowing emotional stimuli to reach awareness rapidly (Vuilleumier et al. 2003; Killgore and Yurgelun-Todd 2004; Phillips et al. 2004; Pourtois et al. 2005; Dehaene et al. 2006; Bocanegra and Zeelenberg 2009; Tamietto and de Gelder 2010; West et al. 2010).

Research on the neurophysiology of the visual system has identified two neuroanatomically defined visual pathways that convey visual information from the retina to the relevant brain areas. These two parallel afferent pathways, magnocellular and parvocellular (also called M and P), project to distinct layers of the lateral geniculate nucleus (Breitmeyer 1984; Merigan and Maunsell 1993; Ogmen 1993). The M pathway is composed of large, rapidly conducting neurons that are specialized for processing rapidly changing stimuli and project to fast-responding areas such as the prefrontal cortex (Bar et al. 2006) or the amygdala (Vuilleumier et al. 2003). The P pathway, on the other hand, is composed of smaller, more slowly conducting neurons that are specialized for processing slowly changing, clearly defined patterns and project primarily through the ventral visual stream to the visual cortex (Merigan and Maunsell 1993; Schechter et al. 2003). A key feature that determines M and P neurons' response properties is spatial frequency (Legge 1978; Tootell et al. 1988; Slaghuis and Curran 1999; Kaplan 2005). M neurons are strongly activated by stimuli that are relatively large (low spatial frequency; LSF) and are involved in initial detection and segregation of objects from the background and in providing gross information about shape. Conversely, P neurons are activated by relatively small (high spatial frequency; HSF) stimuli and code the details of objects (Merigan and Maunsell 1993; Butler et al. 2001).

By manipulating the spatial frequency of visual stimuli, investigators have examined the interplay between basic visual processing and facial affect perception (Vuilleumier et al. 2003; Pourtois et al. 2005; Bocanegra and Zeelenberg 2009). For example, studying the effect of emotion on early visual perception, Bocanegra and Zeelenberg (2009) demonstrated that emotional priming facilitated perception of LSF stimuli, yet inhibited perception of HSF stimuli. They interpreted the LSF benefits as consistent with the idea that emotion enhances magnocellular processing (Bocanegra and Zeelenberg 2009). Pourtois et al. (2005) examined psychophysical responses to filtered photographs displaying facial expressions. They found that LSF emotional information, unlike HSF information, produced early evoked potentials, suggesting a visual pathway that is preferentially tuned to coarse magnocellular inputs of emotional expression (Pourtois et al. 2005). Vuilleumier et al. (2003) employed a gender identification task to compare event-related fMRI responses to unfiltered broadband spatial frequency (BSF) or filtered HSF and LSF faces displaying a fearful or neutral expression. Neural responses in fusiform cortex were greater with HSF facial stimuli, regardless of emotional expression, whereas amygdala responses were greater to fearful LSF faces (Vuilleumier et al. 2003). Furthermore, they reported a differential activation of the pulvinar and superior colliculus by LSF fearful expressions, suggesting a subcortical fear-related LSF input to the amygdala. Thus, it appears that the M pathway has relatively direct projections to subcortical regions such as the amygdala and ventral striatum, enabling faster processing of coarse emotional LSF information, whereas the fusiform cortex, receiving primarily P-pathway input, processes the slower, fine-grained HSF visual information about faces in general.

Taken together, these findings suggest a differential involvement of LSF and HSF information in the perception of facial emotional expressions. Specifically, if emotionally relevant LSF information is processed by the rapidly conducting M neurons to fast-responding brain areas, it should be processed more rapidly than similar HSF information. Although this hypothesis is consistent with the literature, it has not been tested directly. This study was an effort to examine the roles of spatial frequency information and temporal processing in the perception of emotional facial expressions. Specifically, we sought to understand how the speed of facial emotion processing varies as a function of spatial frequency composition of facial stimuli.

To address this question, we employed an emotion identification task with spatial frequency filtering, using methods similar to those used in previous studies (Vuilleumier et al. 2003; Pourtois et al. 2005). Importantly, the temporal processing of emotion perception was examined by suppressing visual perception with a single-pulse transcranial magnetic stimulation (TMS), delivered to the visual cortex at six intervals prior to (forward masking) or following (backward masking) stimulus presentation. In TMS, a bank of capacitors is rapidly discharged into an electric coil to produce a magnetic field pulse. When the coil is placed near the head, the magnetic field induces an electric field in the underlying region of the brain, which, when sufficiently intense, depolarizes cortical neurons, generating action potentials (Barker and Jalinous 1985). Such stimulation is a safe way to temporarily alter cortical function, and over the recent years, this methodology has become a standard procedure for investigating perceptual and cognitive functions (Amassian et al. 1989, 1993; Corthout et al. 1999, 2002, 2003; Lamme and Roelfsema 2000; Pascual-Leone and Walsh 2001; Antal et al. 2002).

Given the critical involvement of LSF information in processing emotional expressions, we predicted that participants will perform significantly better in the BSF (containing both frequencies) and LSF emotion identification conditions than in the HSF condition. Additionally, as LSF information is expected to propagate more rapidly through M pathways, than the slower, P-pathway-dependent HSF information, we predicted that in the BSF and LSF conditions visual suppression with TMS will be stronger in the forward than backward masking component, whereas in the HSF condition visual suppression will be stronger in the backward than forward masking component.

## Methods

### Participants

This study included 27 participants (78% men). Mean age of the sample was 41.8 (SD = 7.93; range = 23–55) and mean education was 14.3 (SD = 1.79; range = 10–16). They were recruited through newspaper and online advertisements as a healthy comparison group for a study on early visual processing in schizophrenia. Participants were excluded if they had any of the following: (1) an identifiable neurological condition, (2) evidence of IQ < 70, 3) histories of any psychotic disorders, any diagnosis in the schizophrenia spectrum, recurrent major depression, bipolar disorder, substance dependence, or substance abuse in the past month, and (4) family history (first-degree relatives only) of psychotic disorders. All participants showed corrected visual acuity of at least 20/30 and gave written informed consent after receiving a full explanation of the research according to procedures approved by the Institutional Review Board of UCLA.

### Equipment

Transcranial magnetic stimulation was delivered by a Magstim Rapid magnetic stimulator (Magstim, Inc., Whitland, U.K.), which produces biphasic pulses using a circular coil with a diameter of 9 cm. The coil was always held at a 90 degree angle, perpendicular to the meridian along the sagittal plane of the subject's skull (Corthout et al. 1999; Antal et al. 2002). The bottom of the coil was placed tangential to the curve of the skull on the spot of interest along the grid. TMS intensity was held constant at 70% of the maximum stimulator output.

### Procedures

Two TMS procedures were conducted: a “hotspot” procedure and an emotion identification procedure. The hotspot procedure was designed to empirically determine the optimal positioning of the TMS coil to identify the location of maximal visual suppression. Once the optimal positioning of the coil was determined, we maintained the TMS coil at that location for collecting data throughout the second procedure, involving affect perception.

#### Hotspot procedure

The stimuli for this procedure were letter trigrams that were randomly generated and presented inside a centralized white border. All letters of the alphabet were included, and the letters on the screen were shown in uppercase font (1 degree in height and 2 degrees in width). This task was programmed and run using Presentation software (Neurobehavioral Systems, Inc., Albany, CA). Participants were seated 57 cm away from the computer monitor, and stimuli were presented for 35 msec on a Dell Pentium computer with a 17″ Sony Multiscan 200PS monitor set at 85 Hz screen refresh rate, screen contrast set to 100%, and Brightness set to 66%. Participants responded by pressing the perceived letters on the keyboard.

Before administering the TMS pulse, we adjusted the target threshold for each participant using a staircase procedure (Green et al. 2002; Rassovsky et al. 2004, 2005). In this method, contrast threshold is adjusted to be more difficult if the subject responds with two or three correct letters out of the three letters presented. Conversely, the current contrast threshold is adjusted to be easier to see if the subject responds with 0 or one correct letters out of the three letters presented, thus adjusting the critical threshold to reflect an average of 50% correct. The descending staircase stops after four consecutive reversals at the smallest step, with the critical threshold taken as the average of the last four contrasts where reversals took place. The contrast level was adjusted with contrast values between black (value of 0) and invisible gray (value of 128), but restricted within a linear range of contrast values as established by calibration with a photometer. The contrast that yielded performance at 50% was considered the critical stimulus intensity (CSI) and was maintained throughout the hotspot procedure.

Following CSI determination, participants were asked to complete a series of 12 trials without TMS to assess their baseline accuracy level. Participants were then fitted with a swim cap and a grid that measured 6 cm × 6 cm was drawn over their occipital lobe consisting of rows of squares each 1 cm^2^. The grid started at the inion and went 6 cm up, 3 cm to the left, 3 cm to the right. Participants were shown letter trigrams with a single TMS pulse administered 100 msec after the presentation of the letters. The stimulus onset asynchrony (SOA; the interval between onset of the target and onset of the TMS pulse) for these trials was held constant at 100 msec, because this has been shown to be the optimal SOA for visual suppression (Mulleners et al. 2001). Starting 2 cm above the inion and continuing moving the coil up and down the grid, participants completed 10 trials for each spot until the location for greatest visual suppression (i.e., the spot with lowest accuracy; hotspot) was identified. The coil was positioned at this hotspot throughout the subsequent emotion identification experiment.

#### Emotion identification procedure

The stimuli consisted of black and white still photographs displaying faces with four basic facial emotions (happy, sad, angry, and afraid) derived from the Karolinska Directed Emotional Faces set (KDEF, Lundqvist, D., Flykt, A., and Ohman, A.; Dept. of Neurosciences, Karolinska Hospital, Stockholm, Sweden, 1998). We randomly selected 10 actors (five men and five women) displaying the four different emotions from the KDEF set, resulting in a total of 40 different face stimuli. The face pictures were trimmed to exclude the hair and non-facial contours. This task was programmed and run using e-prime software (Psychology Software Tools Inc., Sharpsburg, PA) and was administered on a Dell Pentium computer with a 17′′ (43 cm) Sony Multiscan 200PS monitor, driven at 160 Hz. Stimuli were presented as dark on a light background. Participants were asked to identify the emotional expression of face stimuli by pressing one of four labeled keys on the keyboard, such that chance level performance was 25%.

**Figure 1:**
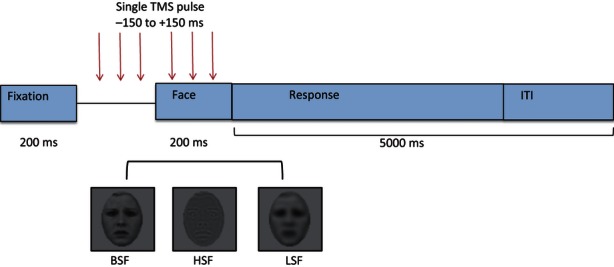
Schematic representation of the study protocol. BSF, broadband spatial frequency; HSF, high spatial frequency; LSF, low spatial frequency.

The face stimuli with BSF was filtered using a high-pass cutoff (≥10 degrees per visual angle) for the HSF face stimuli, and a low-pass cutoff (≤6 degrees per visual angle) for the LSF face stimuli (see Fig. 1). Filtering was performed in Matlab (The Natworks, Natick, MA) using second-order Butterworth filters. High-frequency filtered stimuli bias the system toward M pathways, whereas low-frequency filtered faces bias the system toward P pathways.

The temporal characteristics of affect perception were examined by suppressing visual perception with a single-pulse TMS. Intervals between target and TMS pulse were measured by SOAs, spaced in 50 msec increments from −150 to +150 msec (negative SOAs indicate forward masking, and positive SOAs indicate backward masking). Prior to target presentation, a fixation symbol (a small cross) was presented for 200 msec. The target was presented for 200 msec, with response time and inter-stimulus interval of 5000 msec. These parameters were similar to those used in prior studies of affect perception (Vuilleumier et al. 2003; Holmes et al. 2005; Pourtois et al. 2005). A schematic representation of the protocol is depicted in Figure 1. Participants were seated 1 m away from the computer monitor, and the TMS coil was positioned at the hotspot. To establish a baseline performance, a block of 25 trials without a TMS pulse was administered at the beginning of the procedure. The order of stimuli administration was fully randomized across the 10 actors, four emotions, three spatial frequencies, and seven SOAs (three forward, three backward, and no TMS), with a total of 96 trials per SOA.

### Data analysis

Analyses of variance (ANOVA) with repeated measures were conducted to examine the effects of TMS, spatial frequency, and SOAs. The within-subjects design was structured as a 3 (spatial frequency: high vs. low vs. broad) by 7 (SOAs: −150, −100, −50, 50, 100, 150, no TMS) ANOVA. The primary interest was in the spatial frequency by SOA interaction.

## Results

To validate our hotspot positioning, we compared performance on letter trigram identification with TMS (at 100 msec SOA) against a no-TMS condition with the coil held over the determined hotspot. Pairwise *t*-test analyses revealed that participants performed significantly worse when a single TMS pulse was administered at the hotspot (*M* = 14.3 out of 30, SD = 4.44) than in the no-TMS condition (*M* = 25.3, SD = 2.53), *t*(26) = 12.3, *P* < 0.001. The magnitude of the difference between the means was very large (Cohen's *d* = 3.04).

**Figure 2:**
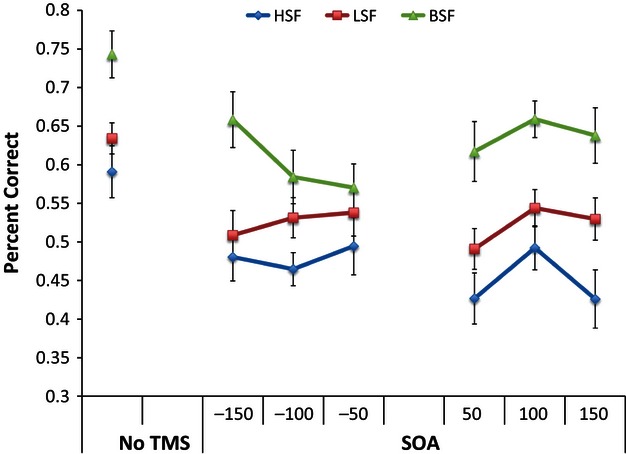
Participants' performance in the different spatial frequency conditions across SOA's. BSF, broadband spatial frequency; HSF, high spatial frequency; LSF, low spatial frequency. ANOVA with repeated measures revealed a significant main effect of spatial frequency (*F*(2,52) = 49.8, *P* < 0.001), SOA (*F*(6156) = 13.4, *P* < 0.001), and a spatial frequency by SOA interaction (*F*(12,312) = 3.19, *P* < 0.001.

Figure 2 presents performance on the Emotion Identification Task. The repeated measures ANOVA revealed a significant main effect of spatial frequency (*F*(2,52) = 49.8, *P* < 0.001), SOA (*F*(6156) = 13.4, *P* < 0.001), as well as a spatial frequency by SOA interaction (*F*(12,312) = 3.19, *P* < 0.001). Pairwise comparisons of the main effect of spatial frequency indicated that in the BSF condition participants performed significantly better than in either the LSF condition (*P* < 0.01) or the HSF condition (*P* < 0.01). Additionally, participants performed significantly better in the LSF condition than in the HSF condition (*P* < 0.05). Pairwise comparisons of the main effect of SOA revealed that participants performed significantly better in the no-TMS condition than in all other conditions (*P* < 0.005), confirming the significant effect of TMS masking across all spatial frequency conditions.

As can be seen in Figure 2, the performance pattern of the three spatial frequency conditions differs more in the forward than that in the backward masking components. Therefore, we repeated the aforementioned analyses separately for the forward and backward masking components. For the forward masking component, there was a significant main effect of spatial frequency (*F*(2,52) = 30.8, *P* < 0.001), and a spatial frequency by SOA interaction (*F*(4104) = 4.45, *P* < 0.005), but no main effect of SOA (*F*(2,52) = 1.98, *ns*). For the backward masking component, there were significant main effects of spatial frequency (*F*(2,52) = 45.5, *P* < 0.001) and SOA (*F*(2,52) = 7.49, *P* < 0.005), but no significant spatial frequency by SOA interaction (*F*(4104) = 1.03, *ns*).

**Figure 3:**
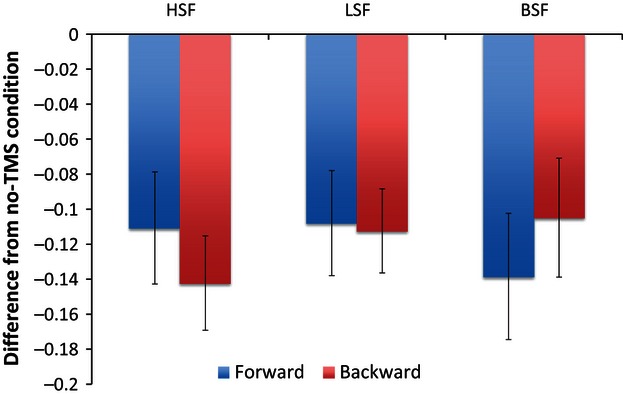
Participants' performance after controlling for baseline (no-TMS) condition and averaging the forward and backward masking components across trials. BSF, broadband spatial frequency; HSF, high spatial frequency; LSF, low spatial frequency. ANOVA with repeated measures revealed a significant spatial frequency by masking condition interaction, *F*(2,52) = 9.25, *P* < 0.001. Pairwise comparisons indicated that in the BSF condition participants performed significantly worse in the forward than backward masking component, *P* < 0.005, whereas in the HSF condition participants performed significantly worse in the backward than forward masking component, *P* < 0.05. No significant differences were detected between the forward and backward masking components in the LSF condition, *P* = 0.74.

To further examine the interaction effect, difference scores were calculated by subtracting each trial from the appropriate baseline (no-TMS) condition (e.g., subtracting HSF trials from the no-TMS HSF condition) and averaging the forward and backward masking components across SOAs. A 3 × 2 repeated measures (spatial frequency by forward/backward masking) ANOVA enabled then an examination of the interaction effect while controlling for baseline performance. These analyses revealed no significant main effects for spatial frequency (*F*(2,52) = 0.23, *ns*) or forward/backward masking (*F*(1,26) = 0.93, *ns*), but there was a significant spatial frequency by forward/backward interaction, *F*(2,52) = 9.25, *P* < 0.001. Pairwise comparisons of the interaction effect indicated that in the BSF condition participants performed significantly worse in the forward TMS masking component than in the backward masking component (*P* < 0.005). Conversely, in the HSF condition participants performed significantly worse in the backward masking component than in the forward masking component (*P* < 0.05). No significant differences were detected between the forward and backward masking components in the LSF condition (*P* = 0.74; see Fig. 3).

**Figure 4:**
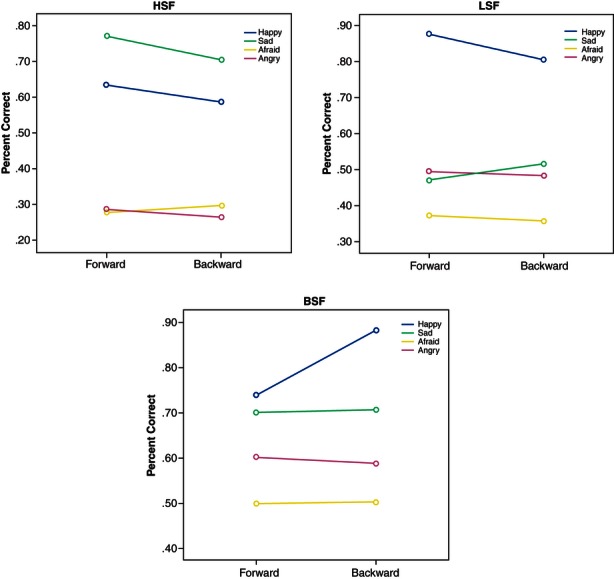
Participants' averaged forward and backward masking performance for each emotion. HSF, high spatial frequency; LSF, low spatial frequency; BSF, broadband spatial frequency. Repeated measures ANOVA revealed significant main effects for spatial frequency (*F*(2,50) = 55.7, *P* < 0.001) and emotion (*F*(3,75) = 56.9, *P* < 0.001), as well as significant spatial frequency by emotion (*F*(6150) = 23.2, *P* < 0.001) and spatial frequency by emotion by forward/backward masking (*F*(6150) = 7.61, *P* < 0.001) interaction effects.

Finally, to examine whether the aforementioned effects were specific to emotion processing rather than face perception in general, we reanalyzed our data by looking at performance accuracy for each of the four emotions. Due to a limited number of trials per emotion (examining the separate emotions was not an original aim of this study), we averaged the forward and backward masking components across SOAs. A 3 × 4 × 2 repeated measures (spatial frequency by emotion by forward/backward masking) ANOVA revealed significant main effects for spatial frequency (*F*(2,50) = 55.7, *P* < 0.001) and emotion (*F*(3,75) = 56.9, *P* < 0.001), as well as significant spatial frequency by emotion (*F*(6150) = 23.2, *P* < 0.001) and spatial frequency by emotion by forward/backward masking (*F*(6150) = 7.61, *P* < 0.001) interaction effects (see Fig. 4). Thus, given the significant variability across emotions, the aforementioned findings are unlikely due to general face perception effects, which are expected to be constant across the different emotions, but rather reflect differences in emotion processing.

## Discussion

This project was an effort to understand how the speed of facial emotion processing varies as a function of spatial frequency composition of facial stimuli. We tested two hypotheses: (1) Given the critical role played by LSF information in emotional processing, we predicted that participants will perform significantly better in the BSF (containing both frequencies) and LSF emotion identification conditions than in the HSF condition. (2) As LSF information is expected to propagate more rapidly through M pathways, than the slower, P-pathway-dependent HSF information, we predicted that in the BSF and LSF conditions visual suppression with TMS will be stronger in the forward than backward masking component, whereas in the HSF condition visual suppression will be stronger in the backward than forward masking component.

Consistent with our first hypothesis, we found that in the BSF condition participants performed significantly better on the affect identification task than in either the LSF condition or the HSF condition, and that the LSF condition yielded better performance than the HSF condition, thereby underscoring the essential role of LSF information in emotional processing. Interestingly, we also found a significant interaction of spatial frequency by SOA effect. Visual inspection of Figure 2 suggested performance differences among the three spatial frequency conditions and SOAs when considering the forward and backward TMS masking components. We examined these differences by first testing the spatial frequency and SOA factors separately for the forward and backward masking components, and subsequently testing the spatial frequency by forward/backward masking interaction effect, after controlling for baseline performance. These analyses revealed two sources for the significant interaction effect. One was that the performance pattern in the BSF condition differed from other spatial frequencies in the forward but not backward masking components, and the second was that the overall level of performance for forward versus backward masking differed by spatial frequency. Consistent with our second hypothesis, we found that in the BSF condition participants performed significantly worse in the forward than backward TMS masking component, whereas the opposite pattern was detected in the HSF condition. Contrary to our hypothesis, no significant differences were detected between the forward and backward masking components in the LSF condition (see Fig. 3).

An important methodological contribution of this study was the use of an empirically based technique for TMS coil positioning (Mulleners et al. 2001). Most studies to date have been utilizing a phosphene (gray or white transient clouds or bubbles within the visual field) induction technique for coil positioning. In this technique, the lower edge of the coil is typically positioned 2 cm rostral to the upper edge of the inion, and the intensity of stimulation is typically set at 80% of each participant's individual V1 phosphene threshold, defined as the TMS intensity where perception of clear stationary phosphenes are perceived 50% of the time (Corthout et al. 1999; Kammer 1999; Pascual-Leone and Walsh 2001; Antal et al. 2002). However, this technique involves substantial degree of subjective judgment on the part of the participant, there is substantial individual variability in the perception of phosphenes, and some participants may not report seeing phosphenes at all (Kammer 2007). Indeed, when conducting preliminary validation of this procedure, we directly compared it with the traditional phosphene method. We found that visual suppression with the Hotspot procedure yields more centrally located hotspots with less variability than the phosphene method. Additionally, we have repeatedly demonstrated during piloting that moving the coil left of center suppressed the right letter of the horizontal trigram, whereas moving the coil right of center suppressed the left letter, thereby suggesting visual suppression of both visual fields.

This study did not include a brain mapping component, limiting our ability to directly determine the neural substrate of stimulation. Future studies employing this procedure would benefit from MRI-based mapping (e.g., co-registering the Hotspot procedure with BrainSight), which would provide information regarding the actual location of visual suppression. Additionally, as we did not have a general face perception condition, we were unable in this study to directly test whether effects were specific to emotion processing versus face perception more broadly. Nonetheless, when examining performance accuracy for the four emotions, we found significant variability across emotions (i.e., significant spatial frequency by emotion by forward/backward masking interaction effect). If the reported effects were due to face perception in general, they are expected to be constant across the different emotions. Therefore, these findings strongly suggest that our results should be interpreted in terms of emotion processing rather than face perception in general. Finally, although in this study we did not include a sham stimulation condition, which limited our ability to control for nonspecific effects of TMS (e.g., clicking noise, scalp and neck muscle twitches), our reliance on empirically based determination of optimal positioning of the TMS coil increased confidence in the results.

To interpret our findings regarding the temporal processing of filtered and unfiltered faces (i.e., the SF by forward/backward masking interaction effect), it would be useful to view these findings within the basic vision framework of the dual-channel model of retino-cortical dynamics (Breitmeyer 1984; Ogmen 1993; Ogmen et al. 2003). An early formulation of this model has postulated that a feedforward mechanism, involving the afferent, unidirectional flow of information from the retina to and through the visual cortex, was sufficient to account for early visual processing (Breitmeyer and Ganz 1976; Breitmeyer 1984). However, data have been accumulating to suggest that the activity of cortical neurons is not determined by this feedforward sweep alone (Enns and Di Lollo 2000; Lamme and Roelfsema 2000; Lamme et al. 2000; Wokke et al. 2013). Instead, conscious visual processing appears to require iterative feedforward–feedback reentrant exchanges of neural signals among levels (Hupe et al. 1998; Di Lollo et al. 2000; Pascual-Leone and Walsh 2001; Rassovsky et al. 2005). Reentrant processes, which have become a major focus in cognitive science, are thought to occur as ascending and descending pathways form an iterative loop, so that ascending stimuli would be influenced by descending top-down activity through this process (Di Lollo et al. 2000; Lamme and Roelfsema 2000; Breitmeyer et al. 2004).

Studies examining visual suppression through single-pulse TMS suggest that forward masking reflects the suppression of the early responses in V1 activating the cortical feedforward sweep, whereas backward masking reflects mostly the later V1 responses due to reentrant activation from post-V1 levels (Corthout et al. 1999; Lamme and Roelfsema 2000; Breitmeyer et al. 2004; Wokke et al. 2013). Consistent with other TMS studies of early visual information processing (Corthout et al. 1999), in this study BSF face stimuli were suppressed more with forward than backward TMS masking, suggesting greater reliance on the feedforward process. The filtered HSF faces, on the other hand, were most strongly suppressed in the backward masking components, potentially demonstrating the increasing involvement of reentrant activation from post-V1 levels (Corthout et al. 1999; Breitmeyer et al. 2004).

It should also be noted that the TMS pulse delivered to the visual cortex primarily affects visual processing of M and P neurons at cortical levels (Amassian et al. 1989; Pascual-Leone and Walsh 2001; Antal et al. 2002). However, there is evidence to suggest that emotional processing is also subserved by subcortical M activity directly from the retina to the superior colliculus and then through the pulvinar to the amygdala (Dolan 2002; Vuilleumier et al. 2003; Tamietto and de Gelder 2010; de Gelder et al. 2011). Hence, face stimuli that contain HSF information (i.e., BSF and HSF conditions) capitalize on cortical processing and hence demonstrate a differential reliance on feedforward (BSF) and reentrant (HSF) processes when suppressed with TMS. LSF face stimuli, on the other hand, rely to a much greater extent on subcortical than cortical processes. Therefore, although there were some general effects of TMS on LSF processing in both the forward and backward components, there was no overall difference between forward and backward TMS masking.

Finally, it is possible that intact BSF faces have an inherent perceptual advantage and hence benefit from faster temporal processing. Indeed, as described above, Vuilleumier et al. (2003) have demonstrated dissociation between fast subcortical LSF emotional processing and cortically mediated perception of HSF facial information. In line with this view, subcortical regions, such as the amygdala and ventral striatum, could provide the necessary (but not sufficient) coarse emotional LSF information that is being complemented by the fine-grained HSF information subserved by the fusiform cortex. In this manner, a quick and efficient perceptual processing of facial emotion information is afforded only when the broad band of spatial frequencies is intact.
